# Checkpoint inhibitor/interleukin‐based combination therapy of cancer

**DOI:** 10.1002/cam4.4659

**Published:** 2022-03-17

**Authors:** Keywan Mortezaee, Jamal Majidpoor

**Affiliations:** ^1^ Department of Anatomy, School of Medicine Kurdistan University of Medical Sciences Sanandaj Iran; ^2^ Department of Anatomy, Faculty of Medicine, Infectious Diseases Research Center Gonabad University of Medical Sciences Gonabad Iran

**Keywords:** combination therapy, immune checkpoint inhibitor, interleukin, programmed death‐1 receptor, programmed death‐ligand 1

## Abstract

**Background:**

Immunotherapy using immune checkpoint inhibitors (ICIs) is the current focus in cancer immunotherapy. However, issues are raised in the area, as the recent studies showed that such therapeutic modality suffers from low durability and low or no efficacy for patients with some tumor types including cases with non‐inflamed or cold cancers. Therefore, efforts have been made to solve the issue using immune combination therapy, such as the use of immunocytokines. The combination of ICI with interleukins (ILs) and IL‐targeting agents is now under consideration in the area of therapy, and the primary results are promising.

**Purpose:**

The focus of this review is to discuss the possibility of using ILs and IL‐targeting drugs in combination with ICI in cancer immunotherapy and describing recent advances in the field using PEGylated ILs and fusion proteins. The key focus in this area is to reduce adverse events and to increase the efficacy and durability of such combination therapy.

## Graphical abstract

Exhaustion of CD8^+^ T cells is promoted by high PD‐L1 expression on immune cells within the TME. Exhaustive CD8^+^ T cells can retain their effector function when they are under exposure to adequate stimuli. Solo ICI shows low durability and efficacy, but its combination with an appropriate IL‐based therapy is important for reducing resistance and increasing the efficacy of therapy.

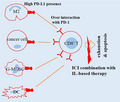

## 1 INTRODUCTION

Immunotherapy is a growing modality in cancer therapy.^1^ The efficacy of cytokine therapy is tested in various preclinical and clinical settings. Immunocytokine therapy directly activates cells of the immune system, such as T cells and natural killer (NK) cells. NK cells, for instance, have receptors for interleukins (ILs) 2, 7, 12, 15, 18, and 21, so IL‐based therapy aiming at potentiating the activity of these cytokines may be effective for enhancing the effector function of NK cells.^2^ A point is that cytokine therapy in its natural form often renders low responses. Short half‐life related to the use of cytokines hampers their therapeutic exposure and efficacy. Activation of counter‐regulatory pathways and the resultant immunosuppression is another burden reducing the efficacy of such therapy.^3^

Another treatment approach is the use of immune checkpoint inhibitor (ICI) therapy, which is a recent focus in cancer immunotherapy.^4^ Engagement between programmed death‐1 receptor (PD‐1) with programmed death‐ligand 1 (PD‐L1) promotes a peripheral tolerance and compromises anti‐tumor immunity.^5^ Targeting interactions between PD‐1 and PD‐L1 is regarded as a breakthrough of the year 2013 in the Science Journal and is honored by Nobel Prize in Physiology and Medicine in the year 2018. Allison and Honjo started a groundbreaking work in the year 1992 in this area and used the term “brakes on T cell activation” for the activity of checkpoints in the tumor microenvironment (TME).^6^ However, only a number of patients develop sustainable responses to the anti‐PD‐1/PD‐L1 therapy.^5^ Microsatellite stability subtype of colorectal cancer (CRC), for instance, shows a limited response to anti‐PD‐1 therapy.^7^ Pancreatic cancer shows high infiltration of immunosuppressive T cells, while prostate cancer represents limited base‐line T cell infiltration, both of which are low responsive to the ICI therapy.^8^ Tumor cells are, in fact, making huge efforts to thwart the efficacy of immunotherapy.^9^ The strategy of combination therapy using ILs and IL‐targeting agents along with checkpoint inhibitors is the focus of this review. Here, studies published in the area of checkpoints and immunocytokine therapy are interpreted in order to uncover more facts about the safety and efficacy of such a combination schedule in cancer immunotherapy. Finding an appropriate combination for ICI therapy in cancer patients is an urgent need of the current years. Knowledge in this area is thus being important for solving the current issues related to the ICI, particularly in patients with non‐inflamed or cold cancers.

## 2 IMMUNE CHECKPOINTS AND CHECKPOINT INHIBITORS

ICI therapy is an approach in cancer immunotherapy that is designed for targeting checkpoint mediators. PD‐1/PD‐L1, cytotoxic T lymphocyte‐associated antigen‐4 (CTLA‐4), and T cell immunoglobulin mucin‐3 (TIM‐3) are known checkpoints used in ICI.^10^, ^11^, ^12^ Pembrolizumab and nivolumab are PD‐1 inhibitors, whereas avelumab, durvalumab, and atezolizumab are PD‐L1 suppressors. Ipilimumab is the inhibitor of CTLA‐4.^13^

### 2.1 The impact of immune checkpoints on cells of tumor immune ecosystem

**FIGURE 1:**
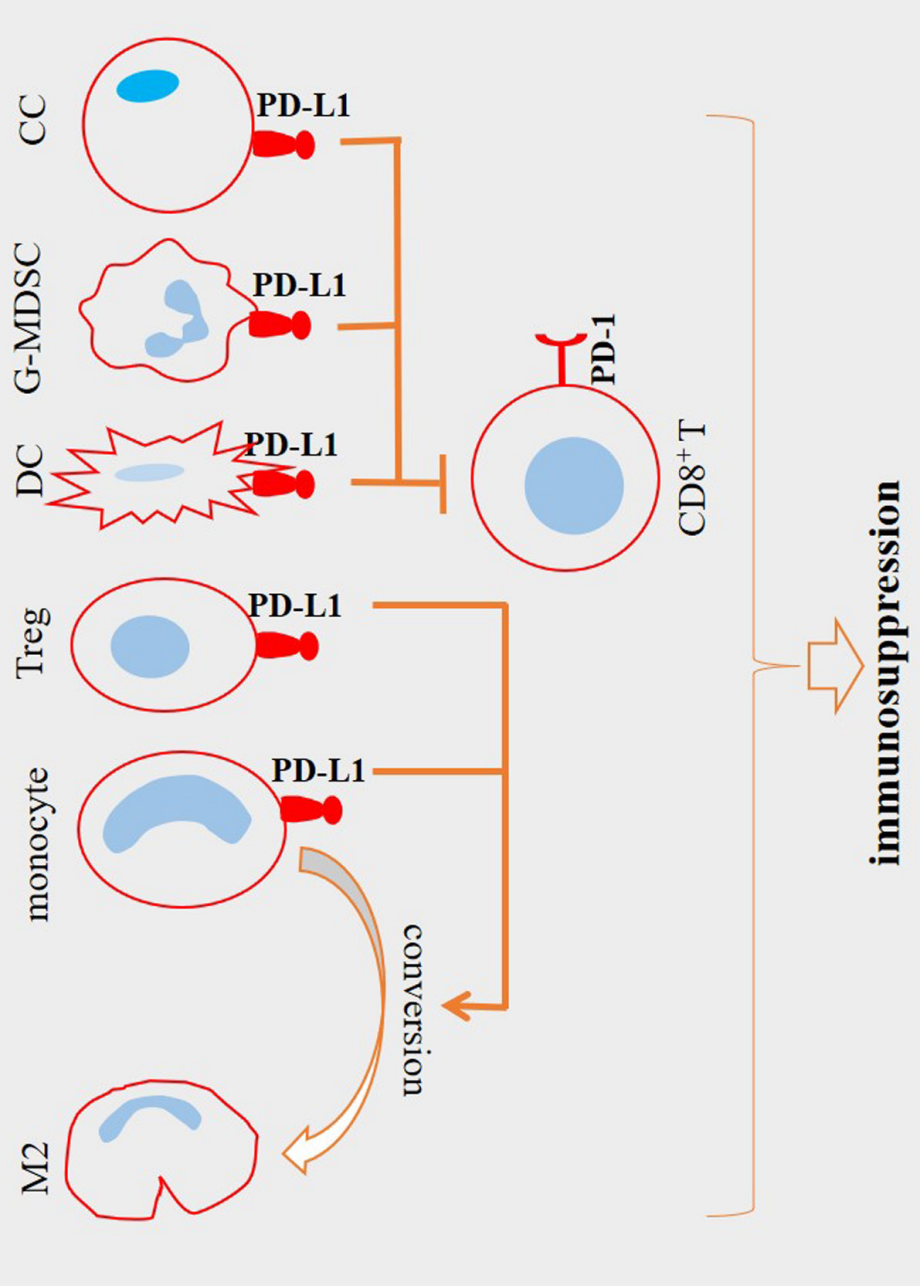
The immunosuppressive activity of programmed death‐ligand 1 (PD‐L1) in tumor microenvironment (TME). Dendritic cells (DCs) express PD‐L1 to suppress responses from CD8^+^ T cells. PD‐L1^+^ tumor cells and PD‐L1^+^ myeloid‐derived suppressor cells (MDSCs) suppress CD8^+^ T cell effector function and promote their exhaustion. PD‐L1 expression is also related to the macrophage polarization toward the pro‐tumor macrophage type 2 (M2) phenotype. This preferential polarity is stimulated by PD‐L1^+^ regulatory T cells (Tregs). PD‐L1 acts via interaction with a programmed death‐1 receptor (PD‐1). High expression of this receptor in a PD‐L1^high^ TME stimulates Treg proliferation and promotes CD8^+^ T cell apoptosis

The immune system plays a vital role in responses from both normal and tumor cells to the therapeutic modalities.^14^ Polarization toward macrophage type 2 (M2) cells is linked positively with PD‐L1 expression.^15^ There is a positive relation between CD206^+^ TAMs with high expression of PD‐L1 in gastric cancer.^16^ PD‐1 is expressed on the surface of dendritic cells (DCs), CD4^+^ T cells, CD8^+^ T cells, and NK cells.^17^ Diskin and colleagues reported a link between PD‐L1 engagement on T cells with tumor immune tolerance. PD‐L1^+^ T cells mediate engagement of PD‐1^+^ macrophages and induce their polarization into an alternative M2‐like phenotype.^18^ CD8^+^ T cells are the critical effector cells and the known front‐line defensive cells against cancer.^19^ The activity of PD‐1 promotes the proliferation of regulatory T cell (Treg) and facilitates apoptosis of CD8^+^ T cells.^17^ PD‐L1 expression on DCs is a key inhibitor of T cell responses,^20^ and its deletion on DCs enhances anti‐tumor responses from CD8^+^ T cells and restricts tumor growth.^21^ Exhaustion of CD8^+^ T cells is promoted by high PD‐L1 expression on myeloid‐derived suppressor cells (MDSCs).^22^, ^23^ The effector function of CD8^+^ T cells is also suppressed by PD‐L1 expression on tumor cells.^24^ CD8^+^ T cell dysfunction occurs upon the interaction of PD‐L1 with PD‐1.^25^, ^26^, ^27^ In tumors like hepatocellular carcinoma (HCC) the fraction of PD‐1^high^ CD8^+^ T cells is increased outstandingly. PD‐1^high^ CD8^+^ T cells express the inhibitory checkpoints CTLA‐4 and TIM‐3^25^ and produce a low amount of IFN‐γ compared with PD‐1^−^ cells.^28^ The inhibitory role of PD‐1 on CD8^+^ T cells occurs not only during chronic inflammation and cancer but also at the time of acute infection^29^ (Figure 1).

### 2.2 Burdens with solo ICI therapy

ICI has revolutionized cancer‐based therapy over the last decade, but there are still a high number of patients who do not respond well to this approach.^30^ Responses to the PD‐1 inhibitor therapy are diminished in patients with low expression of checkpoints on tumor‐infiltrating lymphocytes (TILs). In fact, the proportion of checkpoint positive cytotoxic T lymphocytes (CTLs) is lower for tumors placed in the category of cold, compared to that for hot tumors. Thus, cold tumors show low or no responses to the ICI.^31^

## 3 COMBINATION OF ICIs WITH IL THERAPY

**FIGURE 2:**
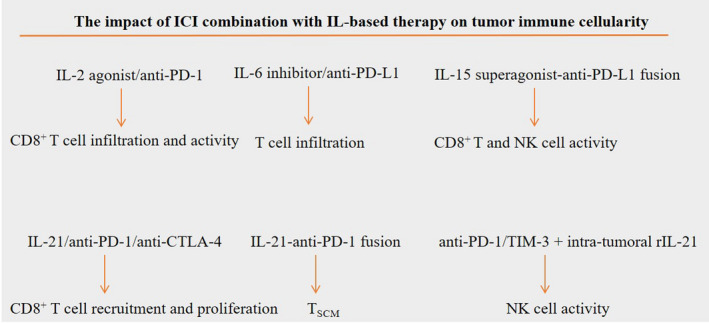
The impact of combinatory immune checkpoint inhibitor (ICI)/interleukin (IL) therapy on cellular immunity within tumor microenvironment (TME). Higher recruitment of CD8^+^ T cells into TME occurs upon a combination of programmed death‐1 receptor (PD‐1) inhibitors with IL‐2 agonist or IL‐6 inhibitors. Bispecific fusion proteins are promising agents in the current research in cancer immunotherapy. N‐809 is an example of such agents that encompasses a fusion complex for IL‐15 superagonist and anti‐programmed death‐ligand 1 (PD‐L1), and it can be used to promote the activity of both natural killer (NK) and CD8^+^ T cells. Fusion of IL‐21 to anti‐PD‐1 antibody promotes the formation of memory stem T cells (T_SCMs_). Effector activity of NK cells is induced by PD‐1/T cell immunoglobulin mucin‐3 (TIM‐3) blockade along with intra‐tumoral rIL‐21. Recruitment and proliferation of CD8^+^ T cells is also induced by IL‐21 when used in combination with anti‐PD‐1 or anti‐cytotoxic T lymphocyte‐associated antigen‐4 (CTLA‐4) antibodies

ICI combination strategy is a promising approach for improving responses in cancer patients.^32^ Cytokines are regulators of the immune system that are active for promoting recruitment of cells of the immune system into TME. They take key roles in cell signaling directed for promoting cellular growth and differentiation, as well as for directing inflammatory or anti‐inflammatory activities. Some cytokines exert potent anti‐tumor activities,^33^ while there are cytokines that act for the promotion of cancer growth and tumor aggressive behavior. Due to the urgent need for controlling TME in patients receiving ICI, cytokine combination therapy with either stimulators or inhibitors is suggested in order to have more potent and durable responses. Some ILs are evaluated in serum samples for predicting responses to the ICI. Thus, assessment of ILs and their application in patients receiving ICI have virtues from both diagnostic and therapeutic viewpoints. The impact of combinatory ICI/IL therapy on cellular immunity within TME is summarized in Figure 2.

### 3.1 Combination with IL‐2‐based cytokine therapy

IL‐2 is a pleiotropic cytokine that acts on various types of cells of the immune system.^34^, ^35^ High PD‐1 expression is contributed to the CD8^+^ T cell anergy through down‐regulation of IL‐2 release from the cells.^36^ Combination of IL‐2 with ICI is thus effective for reinvigorating CD8^+^ T cell responses in patients with chronic infection or cancer.^34^ Combination of high‐dose IL‐2 with PD‐1/PD‐L1 blockade therapy displays an objective response rate (ORR) of 22.5% and 24% for metastatic melanoma and metastatic renal cell carcinoma (RCC), respectively.^37^ Such effects are considerable when compared with monotherapy of high‐dose IL‐2 for metastatic melanoma (ORR: 13%),^38^ but not for metastatic RCC (ORR: 25%).^39^ Hsu and colleagues evaluated the impact of proIL‐2 therapy on tumor resistance to ICI. They noticed an increase in the fraction of PD‐L1^+^CD45^+^ cells, which helps maximizing the bondage of PD‐L1 inhibitors to PD‐L1^+^ cells and thereby overcoming ICI resistance.^40^ Sharma and colleagues investigated the efficacy of bempegaldesleukin combination with ICI in animal tumor model and noticed the synergizing effects and the benefit of using this IL‐2 prodrug for potentiating the fraction of CD8^+^ T cells and cancer regression.^41^ Bempegaldesleukin (also called NKTR‐214) is an IL‐2 agonist that acts preferentially on CD122 (IL‐2β receptor). The impact of bempegaldesleukin combination with nivolumab on T cells and clinical responses to such therapy has been investigated recently for a number of advanced solid tumors including melanoma, RCC, and non‐small cell lung cancer (NSCLC). Total ORR for such combination therapy was 59.5%, which is outstanding. Cellular analysis showed higher infiltration and effector activity of CD8^+^ T cells, while no enhancement in the fraction of Tregs was reported for such therapy.^42^

### 3.2 Combination with IL‐6 targeted therapy

IL‐6 is a pleiotropic and multi‐tasking cytokine with a pro‐ or anti‐inflammatory activity that is produced in chronic inflammatory conditions and cancer. In the context of cancer, it is contributed to tumor cell proliferation, immune escape, angiogenesis, and invasion and metastasis.^43^ IL‐6 is a predictive biomarker of response to ICI. Kang and colleagues evaluated responses to the PD‐1/PD‐L1 inhibitors in NSCLC patients with different baseline IL‐6 levels. Results showed a higher rate of ORR in patients with low IL‐6 levels (a rate lower than 13.1 pg/ml). Patients with low IL‐6 levels also had longer overall survival (OS) and progression‐free survival (PFS).^44^ IL‐6 secretion from macrophages is considered as predictive of weak prognosis in patients with lung cancer. This is due to the inducible effect of IL‐6 on PD‐1 expression from CD8^+^ T cells and the promotion of M2 polarization. IL‐6 targeting along with anti‐CTLA‐4 therapy has been found to reduce M2 cells and PD‐1^+^ CD8^+^ T cells and further improved the survival of tumor‐bearing mice.^45^ There is also a link between IL‐6 deficiency with upregulation of major histocompatibility complex class I (MHC‐I) and PD‐L1 expression on tumor cells. Anti‐PD‐L1 therapy is effective for suppressing metastatic colonization of CRC only in mice negative for IL‐6 (but not in IL‐6^+/+^ animals).^46^ Targeting IL‐6 can also be effective for enhancing the efficacy of PD‐L1 blockade therapy of HCC.^47^ In addition, changes in plasma IL‐6 levels are correlated with responses from NSCLC to PD‐L1 inhibitors. This is delineated by improved PFS (11 vs. 4 months) in ICI‐treated patients.^48^

PD‐L1 expression on melanoma cells is upregulated upon IL‐6 blockade. Induction of IL‐6 is also evoked by PD‐L1 blockade. Anti‐PD‐1/PD‐L1 therapy prompted the expression of IL‐6 from PD‐1^+^ macrophages.^49^ This indicates the therapeutic benefits of using IL‐6 inhibitors in combination with PD‐1/PD‐L1 inhibitor drugs. High IL‐6 expression occurs in the stroma of pancreatic cancer. Combination of IL‐6 inhibitor with PD‐L1 blockade therapy increases T cell infiltration and enhances OS in mice with pancreatic cancer.^50^ Glioblastoma is a tumor with cold immunity, thus being insensitive to ICI. Combination with IL‐6 blockade is not effective for sensitizing such tumor to ICI (anti‐PD‐1/anti‐CTLA‐4), but neutralization of IL‐6 along with stimulation of CD40 can cause tumor sensitization to ICI. In fact, the expression of CD40 is induced by IL‐6. Ablation of IL‐6 abrogated CD40 expression. Thus, recovery of CD40 using appropriate stimulators can promote anti‐tumor immunity.^51^

IL‐6 inhibition can be an effective approach for managing immune‐related adverse events (irAEs) related to the ICI therapy.^52^ Application of the IL‐6 inhibitor tocilizumab can be a choice for modulation of steroid‐refractory irAEs occurring secondary to the ICI therapy in cancer patients.^53^ This may confer that tocilizumab therapy can be used as a substitute to the corticosteroid therapy in patients receiving ICI, as shown in the results of the case series.^54^ Outcomes of a recent systematic review on multi‐center case series introduced the use of tocilizumab as a therapeutic choice for the management of irAEs occurring following ICI therapy, and the combination of tocilizumab with ICI is proposed as an option for controlling cancer progression as well as for targeting irAEs of ICI.^55^

### 3.3 Combination with IL‐8 targeted therapy

IL‐8 (also called CXCL‐8) is a chemoattractant of myeloid cells that are generated at high levels in many types of solid cancers. IL‐8 promotes the recruitment of immunosuppressive cells, induces angiogenesis, and stimulates epithelial‐mesenchymal transition and tumor metastasis.^56^ IL‐8 is considered as a biomarker of response to the ICI. In a study, melanoma and NSCLC patients were administered with pembrolizumab or nivolumab, and serum IL‐8 was evaluated during the course of therapy. It was found a link between early reduction of serum IL‐8 with prolonged OS in such cases, which is indicative of the importance of IL‐8 calculation for monitoring or predicting clinical responses to the ICI.^57^ In a large retrospective study, 2000 patients with NSCLC, melanoma, and RCC treated with nivolumab therapy were enrolled, and the results showed a negative relation between elevated IL‐8 serum levels with OS. Further analysis revealed that IL‐8 at 23 pg/ml has its threshold to be considered as a response biomarker in patients who were more benefited from ICI therapy.^58^ A link between IL‐8 with poor ICI responses is well‐documented in the recent study by Yuen and colleagues. Here, an inverse relation between high IL‐8 level (in tumor, plasma, or peripheral blood mononuclear cells) with atezolizumab efficacy was identified in patients with metastatic RCC and metastatic urothelial carcinoma. Myeloid cells are the main source of IL‐8, and that high expression of this cytokine is linked with the suppression of antigen presentation machinery. Therefore, outcomes are improved in patients treated with ICI after reversing the negative impact of IL‐8 on myeloid cells and antigen presentation.^59^

Castration‐resistant prostate cancer is a type of tumor rarely responsive to the ICI therapy. Castration causes high expression of IL‐8, which further droves intra‐tumoral recruitment of granulocytic MDSCs (G‐MDSCs). Blockade of IL‐8 signaling in combination with ICI has been found to augment the intra‐tumoral density of CD8^+^ T cells and delayed the occurrence of castration resistance.^60^ Results of a recent systematic review also identified IL‐8 as a poor prognostic biomarker of gastric cancer.^61^ Mesenchymal stem cells (MSCs) are one of the sources of IL‐8 in tumors like gastric cancer. Sun and colleagues in a study evaluated the mechanism of PD‐L1 regulation in gastric cancer. Results showed the inducible effect of MSC‐derived IL‐8 on PD‐L1 expression from cancer cells, which resulted in tumor cell resistance to the CD8^+^ T cell cytotoxicity.^62^ The outcomes of such a study will rationalize the application of IL‐8 inhibitors in combination with ICI for reawaking the immune system against cancer.

### 3.4 Combination with IL‐10‐based therapy

Pegilodecakin is the PEGylated recombinant IL‐10 that is able to induce CD8^+^ T cells and stimulate IFN‐γ, MHC‐I, and MHC‐II.^63^ Niang and colleagues in a study evaluated responses from T cells to pegilodecakin in cancer patients. Pegilodecakin promoted CD8^+^ T cell expansion both within a tumor and in the systemic circulation. It also activated intra‐tumoral CD8^+^ T cells. Pegilodecakin combination with anti‐PD‐1 therapy resulted in more expansion of LAG‐3^+^ PD‐1^+^ CD8^+^ T cells.^64^ The same group in another study evaluated the impact of pegilodecakin combination with nivolumab or pembrolizumab in advanced RCC. Combination therapy resulted in the median PFS of 13.9 months, which is comparable to the median PFS of 1.8 months for mono pegilodecakin therapy.^63^ The authors in their third study evaluated the impact of such combination for a number of advanced solid tumors. Results of this multi‐center phase 1b trial showed manageable toxicities, along with promising ORR, particularly in patients with NSCLC (ORR: 43%) and RCC (ORR: 40%).^65^ Thus, the combination of the IL‐10 PEGylated form pegilodecakin with anti‐PD‐1 therapy is promising for advanced RCC and NSCLC.

### 3.5 Combination with IL‐12‐based therapy

Effective anti‐PD‐1 therapy requires DC‐T cell cross‐talking. Anti‐PD‐1 therapy stimulates T cells to release IFN‐γ. IFN‐γ further induces DCs to secrete IL‐12.^66^ T cells stimulated with IL‐12 express lower levels of PD‐1 but higher levels of IL‐2 and IFN‐γ.^67^ Tavokinogene telsaplasmid is an IL‐12‐based gene therapy in which plasmid DNA is synthesized to encode IL‐12 and is injected via an intra‐tumoral route. Forced expression of IL‐12 using an intra‐tumoral injection of IL‐12 plasmid has found to augmented the number of checkpoint positive CTLs. A combination of IL‐12 plasmid with pembrolizumab (200 mg) is effective for improving ORR (41%) in cold melanoma patients.^31^ Avelumab was used in combination with NHS‐muIL‐12 in mice model of mammary tumor. Monotherapy with NHS‐muIL‐12 stimulated infiltration of CD8^+^ T cells into the tumor area. The combination therapy augmented the proliferation of CD8^+^ T and NK cells, and it was more effective for hampering tumor growth than solo therapy with either avelumab or NHSmuIL‐12.^68^

### 3.6 Combination with IL‐15‐based therapy

IL‐15 is a cytokine with promising activities against tumors of solid organs. IL‐15 shares the common receptor γ chain with some other ILs and represents a pleiotropic activity on cells of innate and adaptive immunity. IL‐15 preferentially acts on NK and CD8^+^ T cells^69^ in which it is known as a canonical NK cell growth factor and a strong CD8^+^ T cell agonist.^70^ It was found that avelumab therapy considerably enhanced cytotoxic activity of NK cells against PD‐L1^+^ tumor cells of triple‐negative breast cancer (TNBC), and the lytic activity was augmented upon stimulation of NK cells with IL‐2 and IL‐15. Thus, such types of ILs can be used for increasing the therapeutic activity of avelumab.^71^

Superagonists are now being developed for IL‐15. ALT‐803 is a superagonist for IL‐15. The application of ALT‐803 in ovarian cancer is effective for enhancing the cytotoxic activity of NK cells.^72^ ALT‐803 is used in combination with the PD‐1 inhibitor nivolumab in metastatic NSCLC patients. Results of this phase 1b trial showed a safety profile and promising clinical efficacy (ORR: 29%) for such therapy.^73^ N‐803 is another IL‐15 superagonist that exerts killing activity against ovarian cancer cells through boosting NK cell expansion and functionality.^74^ N‐809 is a novel fusion protein and a bispecific agent that encompasses a fusion complex for IL‐15 superagonist and anti‐PD‐L1. This bifunctional fusion protein represents the same capacity to bind to the PD‐L1 as monoclonal antibodies against PD‐L1. Exposure of CD8^+^ T cells with N‐809 has been found to improved their cytolytic activity against tumor cells and potentiated their proliferation. NK cells also display a higher expression profile for activating receptors on their surface after exposure to the N‐809.^75^ The efficacy of N‐809 is surveyed in the murine model of TNBC, and the higher survival rate and lower possibility of spontaneous lung metastasis were the outcomes.^76^ However, clinical trials investigating the efficacy of this bispecific agent are rare, prompting a necessity for its application in various solid tumors in order to know more about its safety and efficacy profile in human subjects.

### 3.7 Combination with IL‐21‐based therapy

IL‐21 is a strong mitogen and survival factor for NK and T cells. The activity of this cytokine also antagonizes the differentiation of Tregs.^3^ Combination of ICI with recombinant IL‐21 (rIL‐21) is a strategy for enhancing the tumor suppressor effect of ICI therapy in tumors with low/no expression of MHC‐I. The efficacy of PD‐1/TIM‐3 blockade along with intra‐tumoral rIL‐21 injection is exploited in MHC‐I deficient mouse model and augmented anti‐tumor effects were related to the enhanced effector activity of exhausted NK cells.^2^ Combination of IL‐21 with anti‐PD‐1 or anti‐CTLA‐4 therapy in animal tumor models resulted in the augmented intra‐tumoral infiltration of CD8^+^ T cells, along with their increased proliferation and a higher proportion of effector memory T cells.^77^

Protein engineering is a strategy for improving the drug‐like efficacy of natural cytokines. Fusion proteins can be used for this aim. Fusion of IL‐21 cytokine mutein to the PD‐1 antibody can improve the serum half‐life of this cytokine. Such a strategy can also be effective for minimizing the detrimental effects of IL‐21 on local antigen‐presenting cells (APCs).^3^ IL‐21/PD‐1 antibody fusion (PD‐1Ab21) promoted the formation of memory stem T cells.^78^ PD‐L1 expression is also suppressed by IL‐21/IL‐21R.^79^ Contrary to this is a study by Zhao and colleagues who recently identified a positive relation between higher IL‐21‐related inflammation in TME with a possibility of Treg‐mediated immune escape via promoting PD‐1/PD‐L1 activity.^80^

## 4 CONCLUSIONS AND FUTURE DIRECTIONS

**FIGURE 3:**
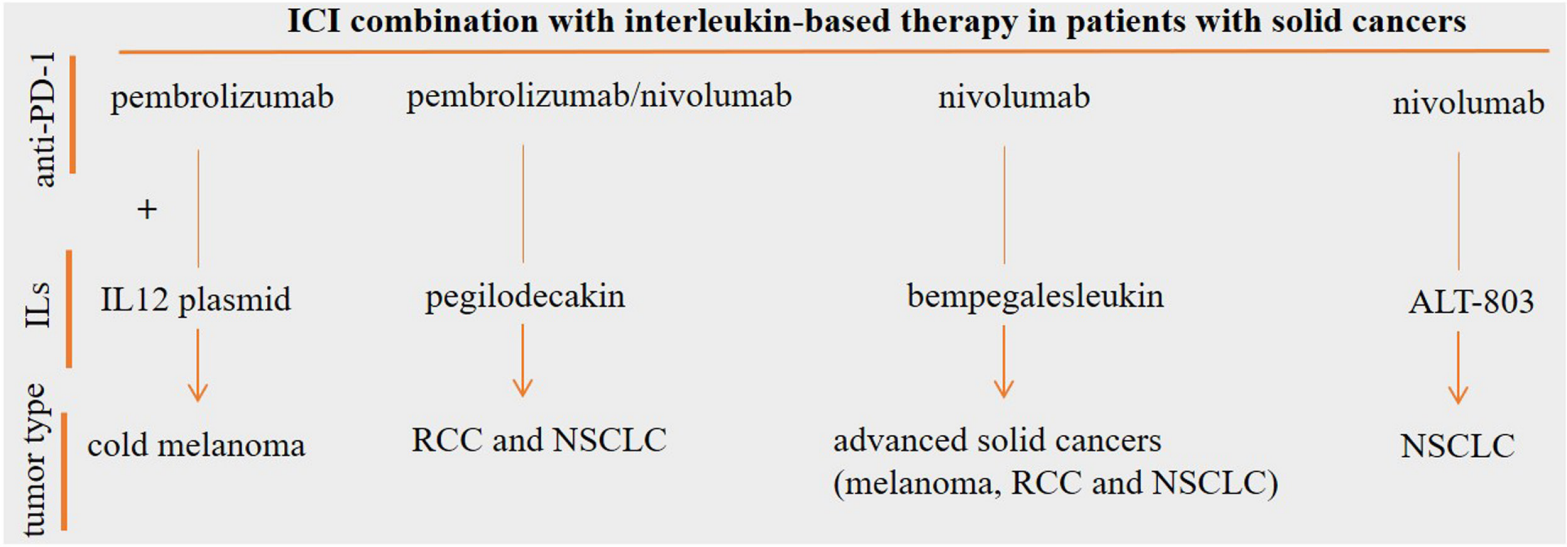
Promising objective response rate (ORR) for combinatory immune checkpoint inhibitor (ICI)/interleukin (IL) therapy in patients with cancer. The combination of programmed death‐1 receptor (PD‐1) inhibitors with IL12 plasmid and PEGylated recombinant IL‐10 (also called pegilodecakin) renders noticeable ORR in cancer patients. ORR is also significant for the IL‐15 superagonist ALT‐803. The most considerable objective response is for the IL‐2 agonist bempegaldesleukin (also called NKTR‐214), which is designed preferentially for CD122 (IL‐2β receptor)

Durable responses only for a number of patients are a major burden related to the ICI therapy, and low half‐life, low efficacy, and high toxicity are issues with regard to immunocytokine therapy. The combination of IL‐based therapy can be exploited as a promising strategy for enhancing the efficacy of ICI therapy in tumors representing low or no responses to the immunotherapy, rendering promising objective responses for a number of tumors (Figure 3). Issues, however, still exist with regard to the use of ILs in cancer immunotherapy. One of the challenges in the area is the diverse functionalities reported for some ILs. IL‐10, for instance, can take pro‐ or anti‐tumor activities. Such diverse activities are due to interactions with different types of receptors. A higher IFN‐γ/IL‐10 ratio is reported in melanoma patients who were responsive to PD‐1 inhibitor therapy.^81^ By contrast, PEGylated IL‐10 is considered a promising combination with PD‐1 inhibitors, as mentioned above. Diverse functionality related to the different types of ILs requires more attention in future studies particularly in a clinical setting in order to inspect whether the combination of immunocytokines with ICIs is an appropriate regimen for patients with different types of cancers or not. There is also an urgent to closely monitor responses from cells of TME to such combination therapy. This will be helpful for uncovering potential counter‐effects related to the therapy. Responses to the ICIs are affected by a number of factors presented in TME, including the rate of TIL infiltration, PD‐L1 expression profile, and tumor mutational burden. Thus, an appropriate combination to the ICI therapy is a drug that works on these variations in favor of therapy, as well as reducing the rate of AEs evolved by patients receiving such combinatory regimen. In the future, more efforts will be made to use PEGylated ILs and fusion proteins in immune combination therapy. What is understood so far is the promising efficacy and acceptable safety profile of such strategy when is used in combination with ICI. However, this area requires more attention, as there are ambiguities and no sufficient proof for many types of tumors in clinical settings.

## CONFLICT OF INTERESTS

The authors declare that they have no conflict of interest.

## AUTHOR CONTRIBUTIONS

Collection and revision of information, Keywan Mortezaee and Jamal Majidpoor; Conceptualization, Keywan Mortezaee; writing, original draft preparation, review and editing, Jamal Majidpoor and Keywan Mortezaee. Two authors have read and agreed to publish the manuscript.

## CONSENT FOR PUBLICATION

The authors of the paper read, approved, and consent to the final version for the publication.

## ETHICS STATEMENT

The manuscript received the Ethical Approval from Kurdistan University of Medical Sciences (Ethical code: IR.MUK.REC.1400.292).

## Data Availability

Data sharing is not applicable to this article as no datasets were generated or analyzed during the current study.
